# One-Shot Laser-Pulse Modification of Bare and Silica-Coated Gold Nanoparticles of Various Morphologies

**DOI:** 10.3390/nano13081312

**Published:** 2023-04-08

**Authors:** Vitaly A. Khanadeev, Andrey V. Simonenko, Oleg V. Grishin, Nikolai G. Khlebtsov

**Affiliations:** 1 Institute of Biochemistry and Physiology of Plants and Microorganisms, Saratov Scientific Centre of the Russian Academy of Sciences (IBPPM RAS), 13 Prospect Entuziastov, Saratov 410049, Russia; 2 Department of Microbiology and Biotechnology, Saratov State University of Genetics, Biotechnology and Engineering Named after N. I. Vavilov, 1 Teatralnaya pl., Saratov 410012, Russia; 3 Saratov State University, 83 Ulitsa Astrakhanskaya, Saratov 410012, Russia

**Keywords:** nanorods, nanostars, nanoantennas, nanoshells, silica shell, photomodification, nanosecond pulsed laser, fluence

## Abstract

Gold nanoparticles are widely used in laser biomedical applications due to their favorable properties, mainly localized plasmon resonance. However, laser radiation can cause a change in the shape and size of plasmonic nanoparticles, thus resulting in an unwanted reduction of their photothermal and photodynamic efficiency due to a drastic alteration of optical properties. Most previously reported experiments were carried out with bulk colloids where different particles were irradiated by different numbers of laser pulses, thus making it difficult to accurately evaluate the laser power photomodification (PM) threshold. Here, we examine the one-shot nanosecond laser-pulse PM of bare and silica-coated gold nanoparticles moving in a capillary flow. Four types of gold nanoparticles, including nanostars, nanoantennas, nanorods, and SiO_2_@Au nanoshells, were fabricated for PM experiments. To evaluate the changes in the particle morphology under laser irradiation, we combine measurements of extinction spectra with electron microscopy. A quantitative spectral approach is developed to characterize the laser power PM threshold in terms of normalized extinction parameters. The experimentally determined PM threshold increases in series were as follows: nanorods, nanoantennas, nanoshells, and nanostars. An important observation is that even a thin silica shell significantly increases the photostability of gold nanorods. The developed methods and reported findings can be useful for the optimal design of plasmonic particles and laser irradiation parameters in various biomedical applications of functionalized hybrid nanostructures.

## 1. Introduction

Gold nanoparticles show great promise for their use in biomedicine due to their unique optical and chemical properties [1,2]. For biomedical applications, the near-infrared wavelength range is most desirable where the so-called biological optical window is located [3]. By changing the material, shape, size, and structure of nanoparticles, as well as their environment, one can tune the spectral position and amplitude of the localized plasmon resonance (LPR) [4]. These properties make it possible to use gold nanoparticles as therapeutic and diagnostic agents [2,5,6].

It was demonstrated that the photomodification (PM) of gold nanoparticles can occur even under laser irradiation parameters that are safe for biological tissue [7]. The shape and size change under the action of laser radiation [8,9]; therefore, the amplitude and position of the plasmon resonance change. All this leads to a decrease in the efficiency of further use of gold nanoparticles as a biomedical agent.

There are a considerable number of articles devoted to the study of the PM of gold nanoparticles of various shapes: nanospheres [10,11,12], nanorods [13,14], nanoshells [15], and nanostars [16]. It has been established that the PM depends on the energy and pulse duration [17,18] and on the solvent in which the particles are located [19]. Depending on the duration of laser pulses, two mechanisms are distinguished: Coulomb instability [20] and photothermal evaporation [21,22]. The Coulomb instability occurs in the femtosecond pulse mode and is associated with the induced emission of photoexcited electrons and strong charge repulsion in ionized nanoparticles. It was also assumed that it could appear in the picosecond pulse mode; however, recently described calculations indicate the opposite [10]. In the case of a nanosecond pulse, the energy acting on the particle lattice leads to its heating to the boiling point of Au [9]. As a result, the size of the nanoparticles decreases.

Most previously reported PM experiments were carried out with bulk colloids, where different particles were irradiated by different numbers of laser pulses. For example, the use of the colloidal solution in a cuvette [17,23,24] may result in multiple irradiations of the same particle, thus leading to an inaccurate determination of the PM threshold. To accurately determine the photomodification threshold, single laser pulses should be used, which has been done in several papers. In particular, it was found [25] that when a colloid of gold nanoshells is irradiated, even at a sufficiently low fluence, the formation of a red spot in the initial green-blue solution can be observed with the naked eye. This color change indicates the formation of small nanospheres from large nanoshells.

To implement a single-pulse laser action, a model was used in which particles move in a fluid jet. Studies were carried out using a cylindrical [26] and a flat [27] liquid jet. It was shown that the latter scheme makes it possible to significantly reduce the nonuniformity of laser irradiation of a liquid volume compared to the cylindrical jet. In addition, X-ray scattering was used to study the effect of a laser on a colloid of gold nanorods when moving through a thin-walled glass capillary [28]. Single-pulse laser treatment was also reported for gold nanoparticles localized on a glass substrate [29]. During the laser-induced splitting of nanoparticles into smaller fragments, craters were formed on the glass surface, the number of which increased with increasing fluence. The single-shot experiments allow the examination of the fluence and laser pulse duration effects.

For laser fragmentation, melting, re-shaping, etc., not only the laser fluence is relevant but also the laser peak intensity (fluence divided by pulse duration). For example, in the case of picosecond and nanosecond laser pulses, the fluence threshold values differ by more than two orders of magnitude [10]. However, when both of these quantities are divided by the corresponding pulse duration, the resulting peak intensity values are similar. As far as we are aware, for gold nanostars, nanoantennas, and SiO_2_@Au nanoshells, a single-pulse PM has not yet been studied.

In this work, we used a scheme of single-pulse irradiation of nanoparticles as they move through a capillary. By controlling the particle concentration, capillary flow, and laser pulse repetition rate, we studied the PM of particles under single-pulsed irradiation. Although the use of a liquid flow setup for laser fragmentation experiments has been reported previously [26,27,28,30,31,32], we present here the first, to the best of our knowledge, detailed comparative study of the single-pulsed laser irradiation of gold nanoparticles of various shapes: nanostars (AuNST), nanoantennas (AuNA), nanorods (AuNR), and SiO_2_@Au nanoshells (AuNSH). We also investigated the effect of silica shells of various thicknesses on the stability of gold nanorods. Finally, we describe a quantitative spectral approach to characterize the PM threshold in terms of normalized extinction parameters.

## 2. Materials and Methods

### 2.1. Chemical Reagents

The following reagents were used without additional purification: Triton X-100, hydrogen tetrachloroaurate(III) trihydrate (HAuCl_4_·3H_2_O, 99%), sodium citrate tribasic dihydrate (Na_3_C_6_H_5_O_7_·2H_2_O, 99%), hexadecyltrimethylammonium bromide (CTAB, 96%), sodium oleate (NaOl, 97%), sodium borohydride (NaBH_4_), hydrochloric acid (HCl), L-ascorbic acid (99.9%), silver nitrate (AgNO_3_, 99.9%), tetrakis(hydroxymethyl)phosphonium chloride (THPC, 80%), sodium hydroxide (NaOH, 98%), l-arginine (98%), potassium carbonate (98%), (3-aminopropyl)trimethoxysilane (97%), tetraethylorthosilicate (TEOS, 98%), ammonium hydroxide solution (30–33%), Tween 20, all purchased from Sigma-Aldrich (Darmstadt, Germany), and thiolated polyethylene glycol (PEG-SH, Mw = 5000, Creative PEGWorks, Chapel Hill, NC, USA). Isopropyl alcohol (IPA) and hydrogen peroxide were purchased from Vekton (Saint Petersburg, Russia). In all experiments, ultrapure MilliQ water (18 mΩ·cm, Millipore, Merck KGaA, Darmstadt, Germany) was used. All glassware and magnets were cleaned with aqua regia (a mixture of HNO_3_ and HCl, in a ratio of 1:3 by volume) and rinsed thoroughly with water.

### 2.2. Synthesis of Gold Nanostars

AuNSTs were synthesized using the seed-mediated two-stage protocol [33], as described in [34]. In the first stage, gold nanospheres with an average diameter of 30 nm were synthesized using the citrate reduction method described by Frens [35] and were used as seeds for the synthesis of gold nanostars. To do this, 244 mL of water was boiled in an Erlenmeyer flask while stirring on a magnetic stirrer. A total of 2.5 mL of 1% HAuCl_4_ and 3.5 mL of 1% sodium citrate were added. The solution was mixed for 15 min. The color of the solution changed from colorless to red. In the second stage, 1 mL of a colloid of 30 nm gold nanospheres, 600 µL of 4 mM AgNO_3_, and 600 µL of 100 mM ascorbic acid were quickly added to a mixture containing 40 mL of water, 1 mL of 1% HAuCl_4_, and 120 µL of 1 M HCl under vigorous stirring. At the same time, the solution quickly acquired a gray-green color.

### 2.3. Synthesis of Gold Nanoantennas

AuNAs were synthesized in the Triton X-100 solution using the seed-mediated method first described in [36] and modified in [37,38]. Briefly, the seeds were obtained by adding 600 µL of a fresh ice solution of 0.01 M NaBH_4_ to a mixture of 10 mL 0.15 M Triton X-100 and 85 µL of 1% HAuCl_4_. Before the synthesis of gold nanoantennas, the seeds were kept for 1 h without stirring. Then, 6 µL of seeds was added to a growth solution containing 10 mL of 0.15 M Triton X-100, 170 µL of 1% HAuCl_4_, 30 µL of 1M HCl, 20.5 µL of 0.788 M ascorbic acid, and 102 µL of 10 mM AgNO_3_. The solution was stirred for 30 min while it acquired a blue-green color.

### 2.4. Synthesis of Gold Nanoshells

AuNSHs were synthesized on highly monodisperse silica cores, as described in [39]. The cores for the gold nanoshells were highly monodisperse silica nanoparticles synthesized as described in [40] based on a combination of two methods [41]: growth in an aqueous medium of l-arginine and overgrowth using the modified Stober method.

Briefly, 9.1 mg of l-arginine was added to 6.9 mL of water in a 10 mL glass vial under magnetic stirring. Then, 0.45 mL of cyclohexane was carefully added to the top of the solution, followed by heating to 60 °C. Furthermore, 0.55 mL of TEOS was dropped into the top layer of cyclohexane and the mixture was gently mixed for 20 h. The average diameter of synthesized silica nanoparticles was about 24 nm. Then, using a twofold similar regrowth procedure, 70 nm silica seeds were synthesized. Specifically, 1 mL of 24 nm synthesized silica nanoparticles was added to 3.6 mL of water. After that, a top layer of 0.5 mL of cyclohexane was applied, and the mixture was heated to 60 °C, followed by the addition of 352 μL of TEOS and stirring for 20 h. The average diameter of the resulting silica nanoparticles was about 45 nm. Then, 1.5 mL of 45 nm fresh silica nanoparticles was added to 3.655 mL of a 0.57 mM L-arginine water solution, followed by the addition of 0.55 mL of cyclohexane (top layer), heating to 60 °C, and the addition of 350 μL of TEOS. The mixture was stirred for 24 h. The average diameter of the synthesized SiNPs was about 70 nm.

For the overgrowth of silica nanoparticles, 300 µL of TEOS was added over 5 h with stirring to a mixture containing 17 mL of absolute ethanol, 1.9 mL of MilliQ water, 0.95 mL of 30% NH_4_OH, and 0.95 mL of 70 nm silica seeds. After the synthesis, silica nanoparticles were washed three times from the reaction products using centrifugation at 18,000 *g* for 30 min and redispersion in ethanol to a final concentration of 7.2 mg/mL.

The next step was to functionalize the silica cores with amino groups. For this purpose, the particles were centrifuged at 18,000 *g* and redispersed in a 4% APTMS solution in ethanol followed by a 24 h incubation. Then, the silica cores were washed using triple centrifugation and redispersion in ethanol.

The next step was the adsorption of gold seeds on the surface of silica cores. Gold seeds with an average diameter of 2 nm were synthesized using the method of Duff [42]. Briefly, 420 µL of 1% HAuCl_4_ was rapidly added to the mixture containing 10 mL of water, 55 µL of 2M NaOH, and 220 µL of THPC solution (12 µL of 80% THPC dissolved in 1 mL of water) under vigorous stirring. Immediately after the addition, the solution turned red-brown and was kept for 24 h. The next day, the pH of the gold seed solution was adjusted to 4 by adding 0.2 M phosphoric acid. A total of 1 mL of aminated silica nanoparticles was added to 10 mL of seeds with pH = 4, followed by 2 h incubation, washing twice using centrifugation, and redispersion in 10 mL of water.

AuNSHs were synthesized by mixing one-day aged 0.9 mM HAuCl_4_ and 0.5 mg/mL K_2_CO_3_ growth solution with 220 µL of seed-decorated silica cores followed by the addition of hydrogen peroxide (0.004%) under moderate stirring for 2–5 min.

### 2.5. Synthesis of Gold Nanorods

The synthesis of AuNRs was based on the seed-mediated protocol described in the article by Murray et al. [43]. In the first stage, gold seeds were synthesized. A total of 600 µL of ice-cold 0.5 mM NaBH_4_ was added to a solution containing 5 mL of 0.2 M CTAB and 5 mL of 0.5 mM HAuCl_4_ under vigorous stirring. The solution was kept for 2 h. To prepare a growth solution, 8.4 g CTAB and 1.481 g NaOL were mixed with 300 mL of water and heated to 50 °C. After that, 21.6 mL of 4 mM AgNO_3_ was added and left for 15 min without stirring. Then, 300 mL of 1 mM HAuCl_4_ was added. The solution was stirred for 90 min, and after that, 2.52 mL of fresh concentrated HCl was added. After another 15 min, 958 µL of 100 mM ascorbic acid and 480.6 µL of seeds were added in turn. The solution was gently mixed and placed in a thermostat for 12 h at a temperature of 28 °C.

### 2.6. Stabilization of Gold Nanoparticles

Before use in biomedical applications, gold nanoparticles are typically coated with PEG-SH. This is done to improve their stability in biological media and to avoid uptake by the reticular endothelial system. Therefore, in this work, we also coated the nanoparticles with PEG-SH and added the secondary stabilizer Tween 20. To do this, 100 µL of 1 mM PEG-SH was added with stirring to 10 mL of a colloid of nanoparticles with pH = 9 adjusted using K_2_CO_3_, left to mix for 30 min, and then 20 µL of Tween 20 was added and stirred for 30 min. After overnight incubation, the nanoparticles were washed from the reaction products using double centrifugation and redispersion in MilliQ water.

### 2.7. Formation of Silica Shell on Gold Nanorods

The silica shell of controlled thickness was formed on AuNRs coated with PEG-SH, as described in [44]. Briefly, the growth of shells with thicknesses of 24, 30, and 57 nm was carried out in a mixture of water and isopropyl alcohol; 95, 150, and 200 mL of isopropyl alcohol were added to 50 mL of the AuNR water colloid, respectively. Next, the required amounts of ammonia and tetraethylorthosilicate (TEOS) were added. To form the 24 nm shell, 8 mL of 3% ammonia and 9 mL of 100 mM TEOS in isopropyl alcohol were added to the mixture under stirring. For the 30 nm and 57 nm silica shells, 4 mL of 3% ammonia was added to each sample followed by the addition of 22.5 and 45 mL of 100 mM TEOS in IPA, respectively, under stirring. The mixture was left overnight at room temperature followed by twofold washing using centrifugation and redispersion in MilliQ water.

### 2.8. Scheme of PM Experiments on Irradiation in a Capillary

**Figure 1:**
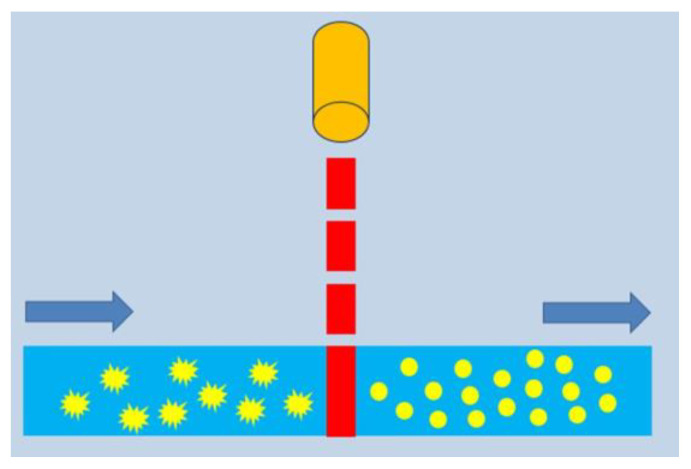
Scheme of pulsed laser irradiation of a nanoparticle colloid moving in a capillary.

To study the PM of gold nanoparticles, we used a scheme with the colloid of nanoparticles moving inside a capillary under pulsed laser irradiation (Figure 1). In a typical experiment, 1 mL of a gold nanoparticle colloid was driven through a 300 micron thick capillary under the pressure of a syringe pump. A tunable nanosecond laser Ekspla NT 200 was used to irradiate nanoparticles perpendicularly to capillary flow. The laser wavelength was tuned to 900 nm, which coincides with the maximum plasmon resonance of AuNRs, AuNSTs, and AuNAs. The laser beam was focused at the capillary middle. The following parameters were used for 900 nm laser irradiation: the linear polarization along the capillary, the pulse frequency of 1 kHz, pulse duration of 2.5 ns, and the length and width of the laser beam focus spot was about 900 μm (perpendicular to the capillary thickness of 300 µm) and 11 µm along the capillary. For laser experiments, nanoparticle colloids were diluted to a gold concentration of about 30–51 mg/mL, thus ensuring the extinction maxima in the range from 0.0284 to 0.0694 for a 300 μm capillary optical path. These conditions correspond to the dilute single-scattering regime.

### 2.9. Fluence Calculation

The fluence values were calculated using the laser radiation parameters specified in Section 2.8. The laser spot had an elliptic shape with the area S=πab, where a=450 μm and b=5.5 μm, thus giving $S=7.7\times 10^{-5}{\mathrm{cm}}^{2}$. Then, using the values of the pulse energy (for example, 1 mJ), we obtained the fluence 13 mJ/cm^2^. Similarly, for a pulse energy *P*(mJ), we obtained the fluence $13\times P\;({\mathrm{mJ}/\mathrm{cm}}^{2})$.

### 2.10. Variation of the Colloid Flow Rate

The colloid movement speed was tuned by a syringe pump, which squeezed out the liquid from the syringe into the capillary. For example, with an extrusion rate of 80 µL/min = 1.33 mm^3^/s and a capillary cross-section area of 0.09 mm^2^, we obtained the linear velocity along the capillary of 1.33/0.09 = 14.8 mm/s. Furthermore, taking into account the width of the laser section along the capillary of 11 µm, the time spent by the particle in the laser beam was found to be 11 µm/14,800 µm/s = 7.4 × 10^−4^ s. The laser pulse frequency was 1 kHz; therefore, at 14.8 mm/s movement speed, some particles could pass through the laser spot without any irradiation. To adjust the irradiation parameters, the extrusion rate from the syringe was set to be 5, 10, 20, 40, and 80 µL/min. These conditions ensured that the particle linear velocity along the capillary was 0.94, 1.88, 3.75, 7.5, and 15 mm/s, respectively. By taking the size of the laser spot and its orientation relative to the capillary at given linear velocities, the particle was irradiated on average by 11.7, 5.9, 2.9, 1.47, and 0.73 laser pulses, respectively. It should be noted that the minimum value of 0.73 laser pulses was chosen based on the balance of two factors: to avoid multiple irradiations of nanoparticles and, at the same time, to be able to irradiate most of the nanoparticles with a single laser pulse.

### 2.11. Extinction and TEM Measurements

Extinction spectra were recorded using Specord 250 and Specord S300 spectrophotometers (Analytik, Jena, Germany). The measurements were carried out in the wavelength range of 320–1100 nm using 2 mm and 10 mm quartz cuvettes. Transmission electron microscopy (TEM) images were obtained using a Libra-120 transmission electron microscope (Carl Zeiss, Jena, Germany) at the Simbioz Center for the Collective Use of Research Equipment in the Field of Physico-Chemical Biology and Nanobiotechnology (IBPPM RAS, Saratov, Russia). For this purpose, the synthesized gold nanoparticles were preliminarily deposited on copper grids.

## 3. Results and Discussion

### 3.1. Gold Nanoparticles

**Figure 2:**
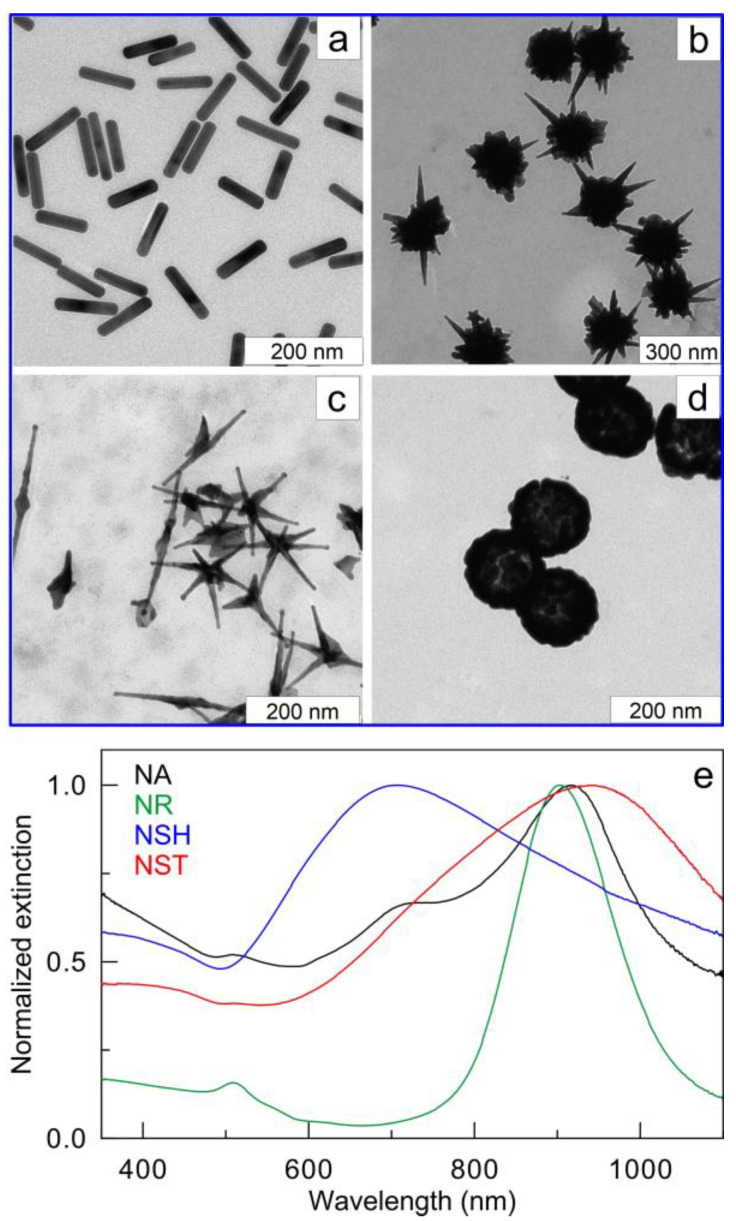
TEM images of AuNRs (**a**), AuNSTs (**b**), AuNAs (**c**), and AuNSHs (**d**) and the corresponding normalized extinction spectra (**e**).

TEM images of fabricated Au nanoparticles are shown in Figure 2. From the TEM examination of 150–200 particle images, the length and thickness of AuNRs are 102.7 ± 7.3 nm and 22.6 ± 1.5 nm, respectively. AuNSTs were obtained with a core diameter of 144.2 ± 12.3 nm and a spike length of 122.6 ± 14.9 nm. The core diameter and spike length of AuNAs were 28.3 ± 2.5 nm and 78.4 ± 10.4 nm, respectively. Finally, for AuNSHs, the diameter of the silica core was 112 ± 3.1 nm, the shell thickness was 14.9 ± 1.8 nm, and the total particle diameter was 141.7 ± 5.2 nm. The main LPR peaks of AuNRs, AuNSTs, and AuNAs were tuned to laser wavelength 900 nm. AuNSHs have a broad LPR resonance ranging from 600 to 1000 nm, with a maximum near 700 nm. It is important to note that AuNSHs also have a rather high extinction value (75% of the maximum) at the laser wavelength of 900 nm.

### 3.2. Silica-Coated Gold Nanorods

**Figure 3:**
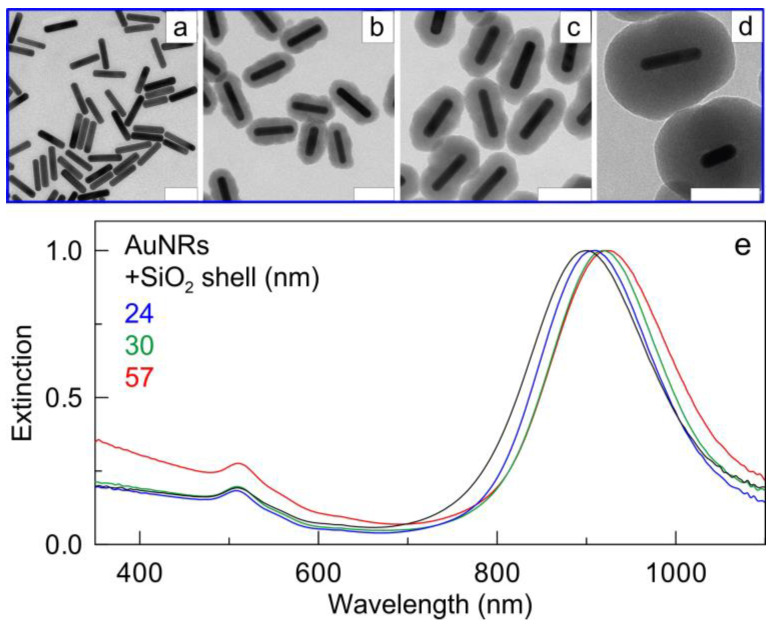
TEM images of AuNRs before (**a**) and after coating with the silica shell of 24 (**b**), 30 (**c**), and 57 (**d**) nm. Scale bars are 100 nm. Normalized extinction spectra (**e**) of AuNRs (black) and AuNR@SiO_2_ composites. Note increased short-wavelength extinction for 57 nm silica shell due to scattering contribution.

Three samples of silica-coated gold nanorods were synthesized, with an average thickness of the silica shell of about 24, 30, and 57 nm. The samples were labeled according to the thickness of the silica shell: AuNR@SiO_2_-24, AuNR@SiO_2_-30, and AuNR@SiO_2_-57. The fabrication of a thinner silica shell (say, less than 10 nm) on the AuNR surface is not an easy task. When a small amount of tetraethylorthosilicate is added, it does not completely cover the rod surface, and the resulting silica shell consists of small fragments, which are often adsorbed at the rod ends. In our experiments, the minimal thickness of the silica shell was 24 nm. It is worth noting that even in this case, the fabricated silica shell had an uneven structure but completely covered the rods (Figure 3b). With an increase in the amount of added TEOS and ammonia, the shell thickness reached 30 nm and became more even (Figure 3c). In the case of a thick shell, so that the total particle size does not exceed 200 nm, a shell with a thickness of 57 nm was synthesized (Figure 3d). The shell surface was much smoother and more spherical than in the case of thinner shells.

Spectral changes during the formation of silica shells are shown in Figure 3e. When a thin 24 nm shell is formed, the main plasmon resonance (PR) localized at 900 nm is shifted by approximately 10 nm towards longer wavelengths as a result of a change in the local dielectric environment of the nanoparticles. With an increase of the shell thickness to 30 nm, the LPR shifts to 920 nm. Finally, for the maximal 57 nm shell thickness, not only a further shift of the PR to 935 nm is observed, but also a significant increase in the short-wavelength shoulder caused by light scattering from the silica shell.

### 3.3. Effects of Flow Rate

**Figure 4:**
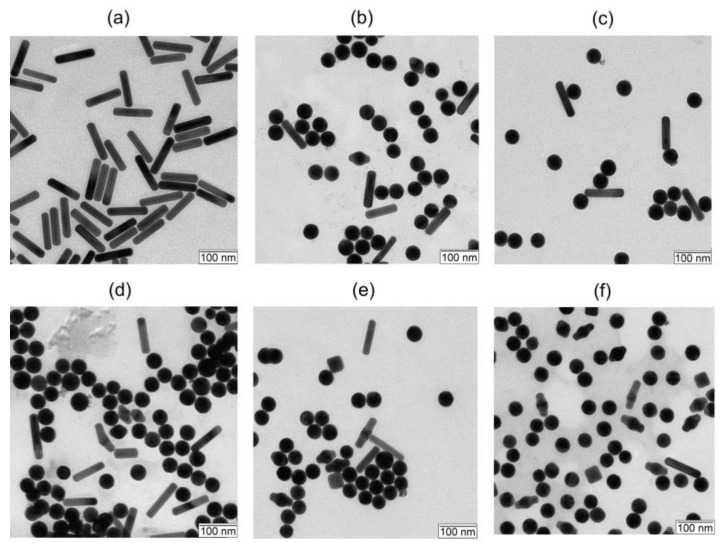
TEM images of gold nanorods before (**a**) and after PM while moving through the capillary at a speed of 15 (**b**), 7.5 (**c**), 3.75 (**d**), 1.88 (**e**), and 0.94 mm/s (**f**), which corresponds to irradiation of 0.73, 1.47, 2.9, 5.9, 11.7 laser pulses, respectively. Scale bars are 100 nm.

First, we examined the effects of the nanoparticle movement in a capillary at different speeds. Our goal was to elucidate the role of multiple laser irradiation for the observed nanoparticle PM. In other words, we investigated how strong the influence of several pulses on the particle change can be. TEM images of gold nanorods before and after PM in a capillary while moving at different speeds are shown in Figure 4. In the initial colloid of gold nanorods, more than 95% of the particles have a rod shape, and the percentage of by-product particles is less than 5%. (Figure 4a). After laser irradiation in a capillary at a speed of 15 mm/s (0.73 laser pulses), there are three fractions of particles in the sample: (1) the initial nanorods; (2) the so-called Φ-particles, which look like a transitional form between the rod and the sphere; and (3) spheres, which are the product of PM of gold rods (Figure 4b). The proportion of gold nanospheres in the samples gradually increases with a decrease in the speed of movement, and the number of initial rods decreases.

These observations are quite expected. Indeed, if one or several pulses hit one particle, it transforms into a sphere because, with a relatively low fluence of 97 mJ/cm^2^, the rods do not split into smaller fragments. The Φ-particles are also present in all samples, and with a decrease in the velocity of the liquid, their fraction gradually increases. The nanorods, Φ-particles, and nanospheres shown in these images have a similar volume. This is explained by the fact that during laser irradiation, nanorods are heated to the melting point of gold and are transformed into Φ-particles or nanospheres. It should be noted that the original rods are also present in all samples (Figure 4). In particular, even at a minimal speed of colloid movement through the capillary (0.94 mm/s (11.7 laser pulses), Figure 4f), we still observe the unmodified AuNRs. This result is somewhat unexpected because at such a low speed the particles are in the laser beam for $5.5\times 10^{-3}$ s and must be exposed to the laser several times. The most probable cause is fluence gradients that occur due to the refraction of the laser on the capillary interface [27]. As a result, more energy enters the central region of the capillary than the peripheral ones. Another possible explanation for this observation is a non-collinear orientation of rods to the electric field vector when the particle crosses the laser spot. It is well known that the light absorption for perpendicular orientation is much smaller than that for longitudinal LPR [4]. As the flow speed decreases, the fraction of intact rods gradually decreases and more rods are transformed into Φ-particles.

**Figure 5:**
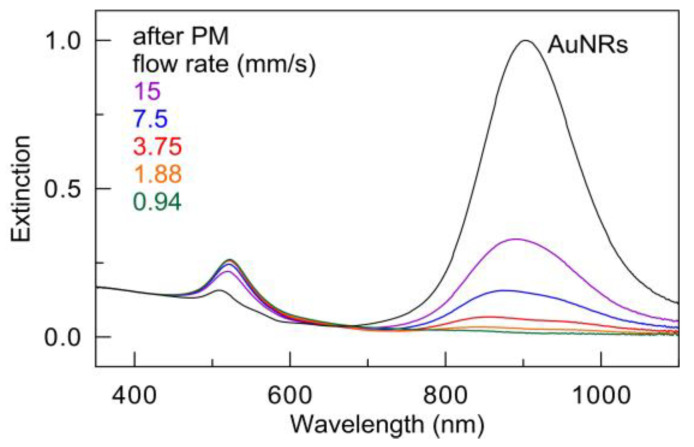
Extinction spectra of a colloid of AuNRs before (black) and after PM using a 900 nm pulsed laser with a fluence of 97 mJ/cm^2^ and when moving through a capillary at a flow rate of 15, 7.5, 3.75, 1.88, and 0.94 mm/s, which corresponds to irradiation of 0.73, 1.47, 2.9, 5.9, and 11.7 laser pulses, respectively.

Figure 5 shows the extinction spectra of AuNR colloids before and after irradiation with a pulsed laser (wavelength 900 nm, laser fluence 97 mJ/cm^2^) at different capillary velocities (0.94, 1.88, 3.75, 7.5, and 15 mm/s), which correspond to the number of laser pulses (11.7, 5.9, 2.9, 1.47, and 0.73, respectively). Here and in the rest of the paper, we used the following normalization procedure. First, we normalized extinction spectra (for a particular nanoparticle type) to their extinction at 400 nm. It has been shown recently [45] that extinction $A_{400}$ gives a universal measure for the molar concentration of gold ($[{\mathrm{Au}}^{0}](\mathrm{mM})=0.44\times A_{400}$) for particles of arbitrary shapes and nanosphere clusters. Furthermore, we normalized all spectra to the major LPR peak of the initial nanoparticles.

A noticeable decrease in the amplitude of the main LPR peak is already observed at the maximum velocity speed of 15 mm/s (0.73 laser pulses), in agreement with the TEM examination of Figure 4b, due to the notable transformation of rods into spheres. This is accomplished by an increase in the intensity of the PR peak at about 530 nm, which corresponds to the LPR of gold nanospheres.

A further decrease in the velocity to 7.5 mm/s (1.47 laser pulses) leads to a further twofold decrease in the long-wavelength PR peak of the rods and a further increase in the PR peak of nanospheres by 530 nm. It is also worth noting the decrease in the quality factor of the long-wavelength PR peak, which is indicative of the polydispersity of particles in the sample. Furthermore, the described trend continues, and at a minimum velocity of 0.94 mm/s (11.7 laser pulses), a typical spectrum of gold nanospheres is observed, while the characteristic longitudinal LPR peak of nanorods disappears. Thus, to avoid multi-pulsed laser impact on particles and to study only the results of single pulse irradiation, a capillary velocity of 15 mm/s (average 0.73 laser pulses per particle) was chosen for further experiments. Furthermore, based on the data in Figure 4 and Figure 5, we conclude that the action of several laser pulses can lead to more pronounced changes in the particle shape of nanoparticles compared to a single pulse PM.

The use of a flat and open jet of a nanoparticle colloid for single-pulse irradiation (see, for example, Zerebecki et al. [27]) makes it possible to overcome the shortcomings of a capillary setup, which causes serious problems of light refraction at the capillary boundary. However, even with the flat jet, some unsolved problems could remain. First, the absorption, heating, and PM of anisotropic particles such as AuNRs strongly depend on particle orientation. Secondly, the spatial distribution of particles can be inhomogeneous because of different particle velocities within the illuminated volume. Finally, the illuminating beam usually has a Gaussian intensity profile rather than that of a plane wave. Thus, even with the flat jet, the beam spot has a strongly inhomogeneous power profile, thus making quite different irradiation conditions for particles located near the beam center and its periphery. Thus, the case of reliable single-pulsed irradiation of particles in a colloid seems rather difficult to implement, in contrast to the case of single-pulse laser action on a single particle localized on a substrate.

To minimize the effect of the Gaussian intensity profile, we used the beam width in the perpendicular direction three times greater than the capillary thickness. Nevertheless, the presence of obviously intact particles in our system after irradiation shows that the level of irradiation of various particles was different. Additional work is needed to more clearly treat the power inhomogeneity or polarization orientation effects.

It should be noted that we did not aim to obtain 100% PM of particles during a single passage. Instead, we tried to irradiate particles with no more than a single pulse, thus avoiding multiple irradiations. Therefore, we selected the appropriate velocity of movement along the capillary in such a way that no more than one pulse fell on one particle. In practice, a greater PM degree can be achieved using various irradiation schemes and flow setups. For example, by using several sequential passages, one could achieve the almost complete PM of particles. However, there is no guarantee that all particles have been irradiated no more than once. In our experimental scheme, we tune the particle velocity, concentration, and repetition pulse rate to ensure one-shot irradiation. On the other hand, some particles we unmodified due to incomplete irradiation conditions.

### 3.4. Effects of Nanoparticle Shape and Structure

Here, were compare the PM of AuNRs, AuNSTs, AuNAs, and AuNSHs at similar experimental conditions: the laser wavelength and LPR peaks are tuned to 900 nm (except for AuNSHs with a broad LPR peak), a capillary flow speed of 15 mm/s (0.73 laser pulses per particle), the laser pulse frequency of 1 kHz, and pulse duration of 2.5 ns. The effects of laser fluence were studied with other experimental parameters being constant.

**Figure 6:**
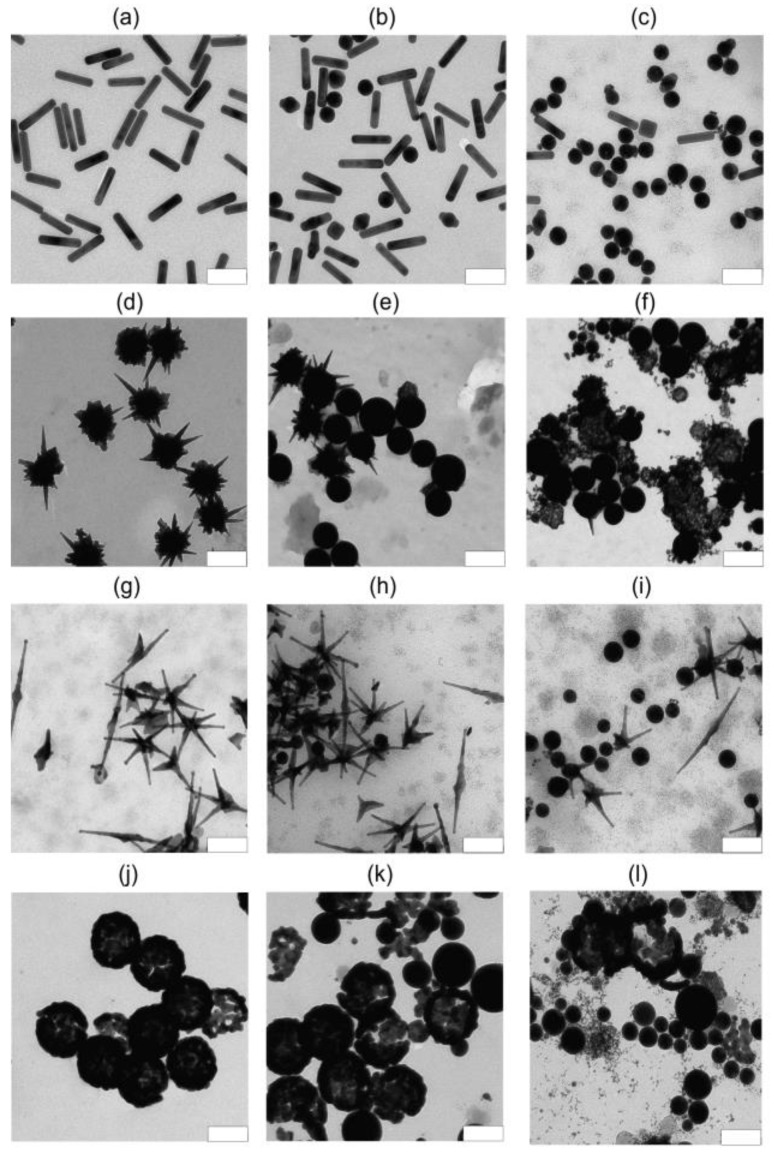
TEM images of AuNRs nanoparticles before (left column) and after (middle and right columns) PM. AuNRs—*F* = 0 (**a**), 40 (**b**), and 520 mJ/cm^2^ (**c**). AuNSTs—*F* = 0 (**d**), 260 (**e**), and 1040 mJ/cm^2^ (**f**). AuNAs—*F* = 0 (**g**), 65 (**h**), and 260 mJ/cm^2^ (**i**). AuNSHs—*F* = 0 (**j**), 130 (**k**), and 520 mJ/cm^2^ (**l**). Scale bars are 100 nm (**a**–**f**,**j**–**l**) and 200 nm (**g**–**i**).

Consider first the morphological changes, as revealed in the TEM images (Figure 6).

For AuNRs, noticeable changes occur starting from a fluence of 40 mJ/cm^2^ (Figure 6b). Fractions of nanospheres and Φ-particles appear in the sample, but the majority of particles are still intact nanorods. When the fluence is increased to 520 mJ/cm^2^ (Figure 6c), most of the particles are gold nanospheres and only a small part of intact rods remains. For AuNSTs, more than half of the particles are transformed into nanospheres at 260 mJ/cm^2^, and the average length of the spikes also shortens (Figure 6e). At a fluence of 1040 mJ/cm^2^, a sharp transformation into nanospheres occurs with the formation of a large number of small gold nanoparticles of various sizes (Figure 6f).

The morphology of AuNAs differs from that of AuNSTs due to the presence of thin long spikes. This is why a noticeable fraction of nanospheres appears already at a fluence of 65 mJ/cm^2^ (Figure 6h), and at a fluence of 260 mJ/cm^2^ (Figure 6i), the number of nanospheres noticeably increases. Nanoshells differ from other particles in that their PM begins with the splitting of the shell into smaller pieces at 130 mJ/cm^2^ (Figure 6k), and as a result, all particles are also transformed into gold nanospheres of various sizes at 520 mJ/cm^2^ (Figure 6l).

**Figure 7:**
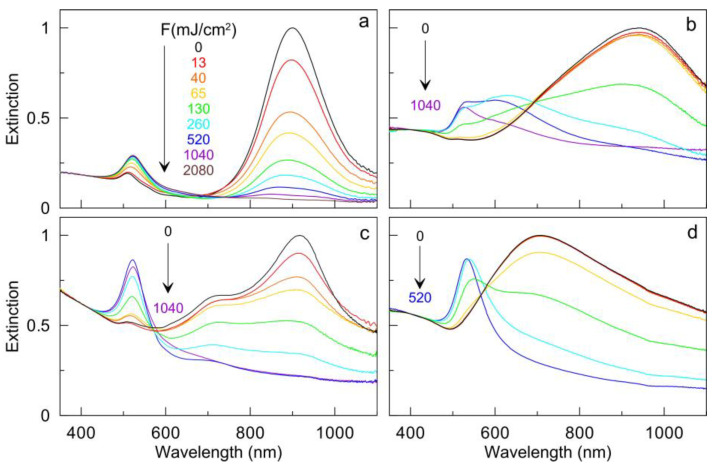
Extinction spectra of AuNRs (**a**), AuNSTs (**b**), AuNAs (**c**), and AuNSHs (**d**) before (black curves) and after irradiation with a pulsed laser with a given fluence while moving through a capillary. The laser fluences (in mJ/cm^2^) are indicated from the top to the bottom.

Spectral analysis allows the convenient monitoring of the ensemble changes in particle morphology. For definiteness, we estimated the fluence threshold value through a decrease in the main plasmon resonance peak by 10%. For AuNRs, an initial PM is observed at a fluence of 13 mJ/cm^2^, the main changes occur in the range of 13–1040 mJ/cm^2^, and almost all particles are photomodified at a fluence of 2080 mJ/cm^2^ (Figure 7a). Accordingly, the amplitude of the longitudinal LPR at 900 nm gradually decreases and broadens, whereas the amplitude of nanosphere LPR at 530 nm gradually increases. For AuNSTs, the threshold value of PM lies near 130 mJ/cm^2^, when the intensity of the main peak noticeably decreases (Figure 7b). The main changes occur in the range of 130–1040 mJ/cm^2^. In addition to the decrease in the main LPR peak, the shoulder at 630 nm increases, which can be explained by an increase in the number of large gold particles with small protrusions on the surface. Furthermore, at a fluence of 1040 mJ/cm^2^, almost all particles turn into gold spheres.

In the case of AuNAs, noticeable changes begin at a small fluence of 13 mJ/cm^2^ (Figure 7c). Notable changes occur in the range of 40 to 520 mJ/cm^2^. Specifically, we observe a decrease in the amplitude of the main PR peak and an increase in the peak at 530 nm. Complete transformation into nanospheres occurs at a fluence of 1040 mJ/cm^2^. For AuNSHs on silica cores, noticeable spectral changes begin at a fluence of 65 mJ/cm^2^ (Figure 7d). Simultaneously, there is a decrease in the main broad PR peak and an increase in the peak at about 540 nm. All changes occur in the range of 65–260 mJ/cm^2^. Finally, at a fluence of 520 mJ/cm^2^, the initial PR maximum completely disappears, and only a peak near 530 nm remains, which is characteristic of spheres. It should be noted that under irradiation below the thresholds of PM, no significant changes in the spectrum and the form of particles occur.

Thus, the PM of all gold nanoparticles evolves according to a similar scenario. From TEM examination, with an increase in the laser fluence, the fraction of initial particles gradually decreases, whereas the proportion of gold nanospheres increases. The spectral changes are also consistent with these observations. Specifically, the amplitude of the main peak of the initial nanoparticles decreases until it disappears completely, whereas the amplitude of the nanosphere peak near 530 nm gradually increases. Our main result of these experiments is that the fluence values for the complete transformation of particles into spheres increase in the series: gold nanoshells (520 mJ/cm^2^)—nanostars and nanoantennas (1040 mJ/cm^2^)—nanorods (2080 mJ/cm^2^). The most sensitive to PM are gold nanorods and nanoantennas, for which the initial PM threshold is observed at a fluence of 13 mJ/cm^2^. For nanoshells and nanostars, the initial PM thresholds are 65 and 130 mJ/cm^2^, respectively.

### 3.5. Effect of the Silica Shell on the Photostability of Nanorods

**Figure 8:**
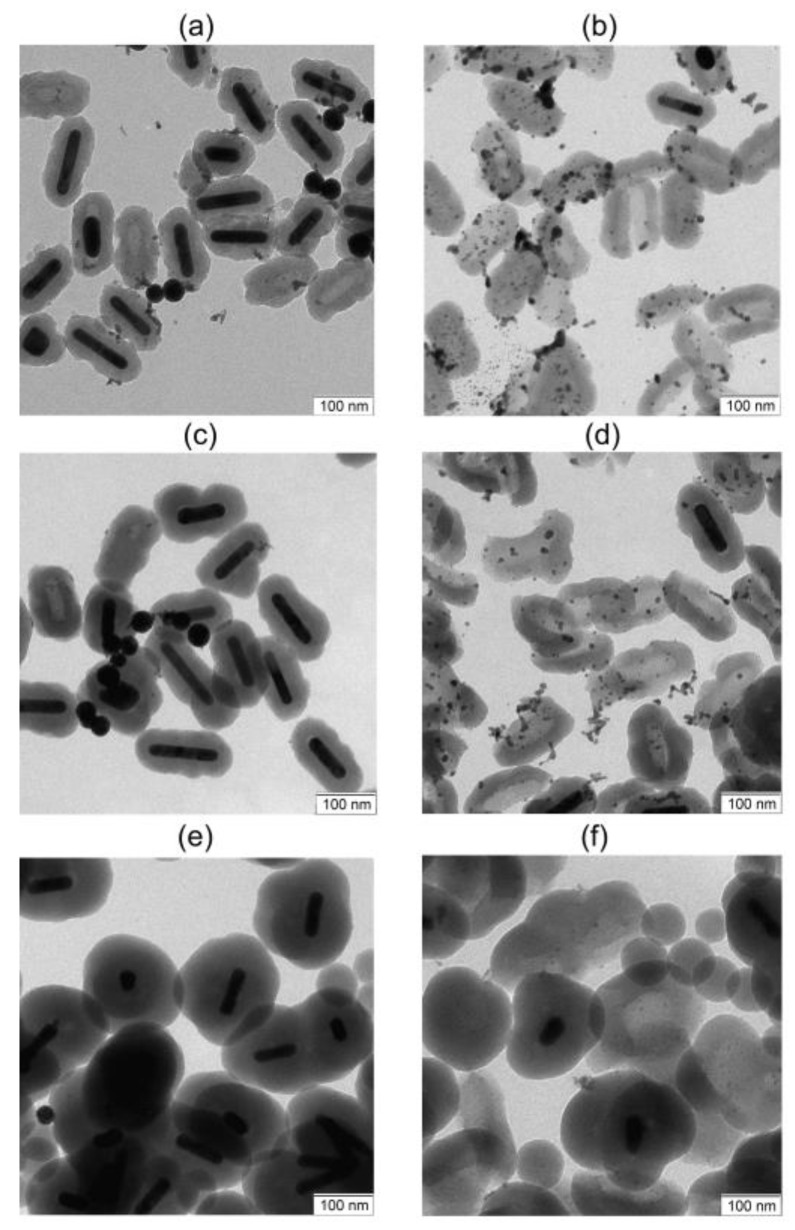
TEM images of nanorods coated with a silica shell after PM while moving through a capillary. Nanorods with a 24 nm shell after irradiation with a fluence of 1040 (**a**) and 4160 mJ/cm^2^ (**b**). Nanorods with a 30 nm shell after irradiation with a fluence of 520 (**c**) and 4800 mJ/cm^2^ (**d**). Nanorods with a 57 nm silica shell after exposure to a laser with a fluence of 520 (**e**) and 4800 mJ/cm^2^ (**f**). Scale bars are 100 nm.

The formation of the silica shell on the Au nanoparticle surface increases its photostability [46] and prevents unwanted changes in plasmonic properties due to particle aggregation. Additionally, the outer silica shell is often used for surface functionalization and drug loading. Consider the morphologic changes in particles in the TEM images (Figure 8). AuNRs with the thinnest 24 nm silica shell (AuNR@SiO_2_-24) are partially photomodified at a fluence of 1040 mJ/cm^2^, and several fractions of particles with different morphology are presented in the sample (Figure 8a).

In some particles, the destroyed shell is observed and AuNRs are transformed into gold spheres, leaving shells with the inner contour of rods. A small fraction of the rods reduces its length and remains inside the shell (one such particle is shown in Figure 8a, left of center). Nevertheless, about half of the rods in the shell remain unchanged. A small amount of small gold nanoparticles is also observed, which are probably formed during the sharp destruction of the initial rods. At a fluence of 4160 mJ/cm^2^, the nanorods are completely transformed into small nanospheres, accompanied by the destruction of the shells (Figure 8b).

In the case of a thicker shell (AuNR@SiO_2_-30), at a fluence of 520 mJ/cm^2^, less than 25% of the rods destroy their shell and transform into nanospheres, while most of the particles remain unchanged (Figure 8c). With an increase in fluence to 4800 mJ/cm^2^, almost all rods are transformed into spheres, while their shells are destroyed (Figure 8d). Most rods with the thickest shell (NR@Silica-57) also retain their structure at a fluence of 520 mJ/cm^2^ (Figure 8e). With an increase in fluence up to 4800 mJ/cm^2^, only a small part of the rods remains inside the shells (Figure 8f), and at a fluence of 5430 mJ/cm^2^, the rods completely transform into spheres.

**Figure 9:**
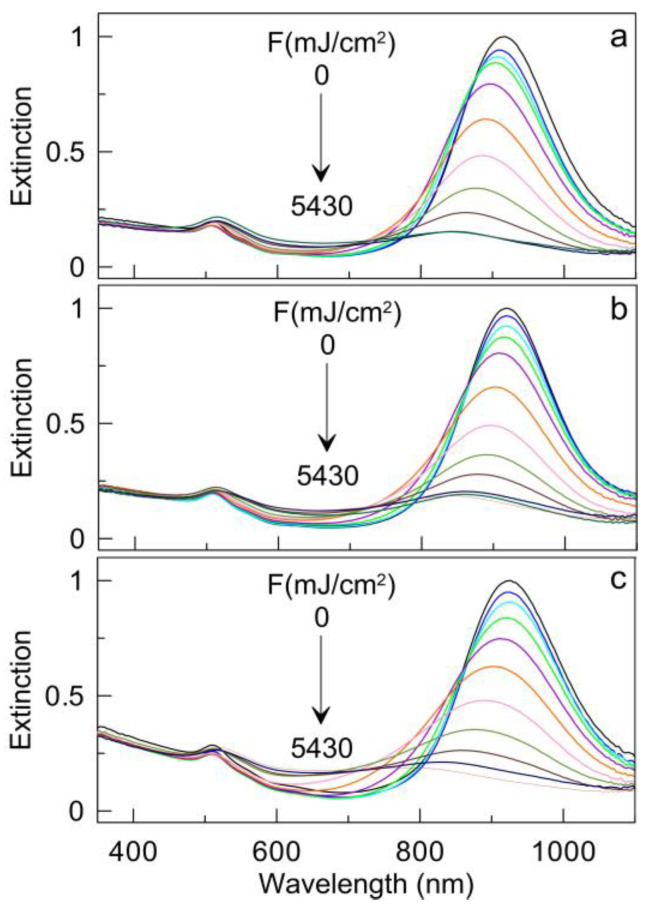
Extinction spectra of gold nanorods coated with a silica shell with an average thickness of 24 (**a**), 30 (**b**), and 57 nm (**c**) before (black curve) and after PM under the action of a pulsed laser with fluence from 13 to 5430 mJ/cm^2^ when moving through the capillary. The fluence values are (from top to the bottom) 0, 13, 40, 65, 130, 260, 520, 1040, 2080, 4160, and 5430 mJ/cm^2^.

The spectral changes are in complete agreement with the above TEM observations. At a shell thickness of 24 nm (AuNR@SiO_2_-24), the threshold PM fluence value equals 65 mJ/cm^2^ (Figure 9a). In this case, the main peak not only decreases, but also slightly shifts towards shorter wavelengths, which may indicate that some of the rods shorten their length, some of the particles lose their silica shell, or both of these processes occur. Furthermore, with an increase in fluence from 130 to 4160 mJ/cm^2^, a gradual decrease and shift of the peak to 850 nm continue. Here, it is worth noting an important difference from the case of rods without a shell (Figure 7a). For coated rods, the LPR intensity near 520 nm does not increase even at high fluence, when all particles are photomodified. This can be explained by the fact that the shell covers the gold rod and prevents its smooth transformation into a large gold sphere. As a result, the shell is destroyed only when the rod abruptly transforms into small gold particles, which have a small extinction cross-section and do not make a significant contribution to the PR peak at 520 nm.

For a silica shell thickness of 30 nm (AuNR@SiO_2_-30), a noticeable initial PM also occurs at a fluence of 130 mJ/cm^2^ (Figure 9b). With a further increase in fluence from 260 to 4800 mJ/cm^2^, similar changes occur in the spectrum: the LPR peak decreases, broadens, and shifts towards shorter wavelengths up to about 850 nm. For the maximum shell thickness of 57 nm (AuNR@SiO_2_-57), PM evolves according to a similar scenario: a noticeable start at 65 mJ/cm^2^ and then a gradual decrease and shift of the main PR to shorter wavelengths (Figure 9c). A characteristic difference here is an increase in the width of the main PR peak. Complete modification occurs at a fluence of 5430 mJ/cm^2^.

Thus, two important conclusions can be drawn from these experiments. First, even the thinnest 24 nm silica shell significantly increases the photostability of gold nanorods. The threshold value for the initial PM rises to 65 mJ/cm^2^ compared to the initial rods (13 mJ/cm^2^), the main changes now occur in the range of 130–4160 mJ/cm^2^ (40–1040 mJ/cm^2^ for the initial rods), and the threshold for the complete PM of particles rises from 2080 to 4800 mJ/cm^2^.

The second conclusion is that the photostability of the particles weakly depends on the shell thickness. Thus, for all three shell thicknesses from 24 to 57 nm, the onset of PM is observed at a fluence of 65 mJ/cm^2^. In the range of 130–4160 mJ/cm^2^, similar spectral changes occur, and differences are observed only in the threshold of complete PM, which increases from 4160 to 5430 mJ/cm^2^ with the increase in shell thickness.

These conclusions are important from a practical point of view as it is sufficient to cover AuNRs with a thin silica shell to significantly enhance their photostability [41].

### 3.6. Quantification of PM Factor

**Figure 10:**
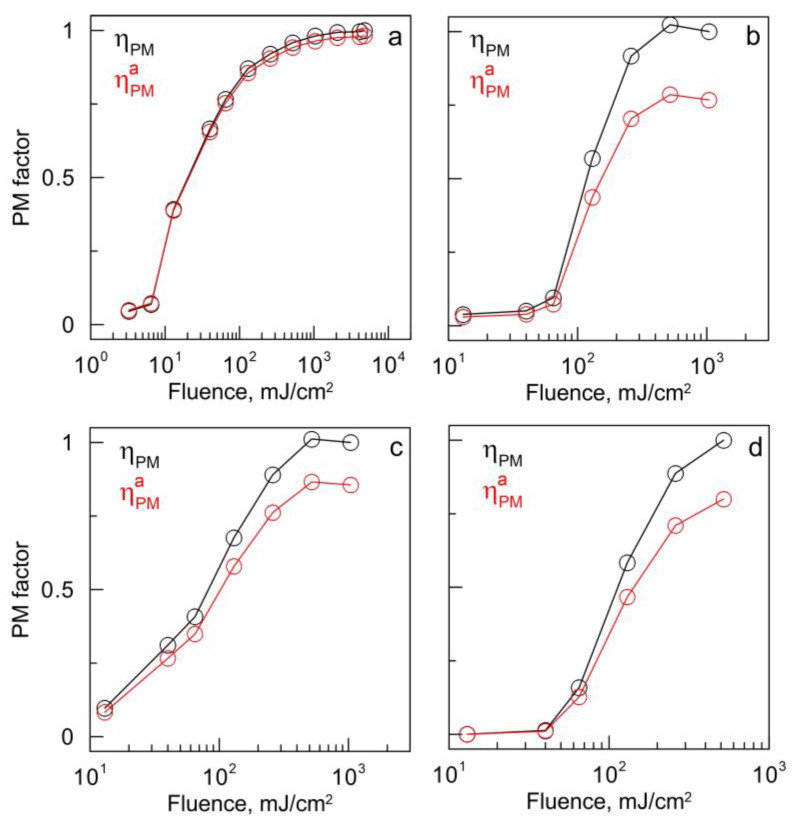
PM factor $\eta_{PM}$ (black curve) and approximate PM factor $\eta_{PM}^{a}$ (red curve) as a function of fluence for four types of gold nanoparticles: nanorods (**a**), nanostars (**b**), nanoantennas (**c**), and nanoshells on silica cores (**d**).

**Figure 11:**
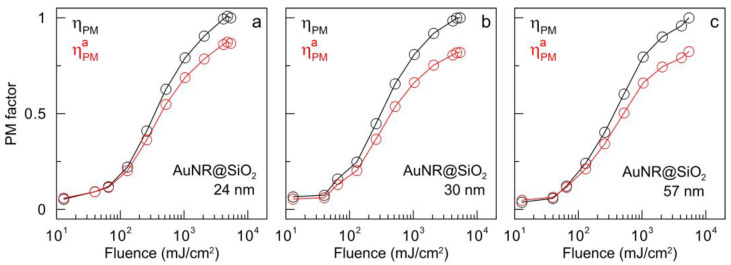
PM factor $\eta_{PM}$ (black curve) and approximate PM factor $\eta_{PM}^{a}$ (red curves) as a function of fluence for gold nanorods with different silica shell thicknesses: 24 (**a**), 30 (**b**), and 57 (**c**) nm.

To quantify the degree of PM, we propose to use the PM factor, which is derived directly from the PM extinction spectra. First, define the normalized extinction parameter:

(1) $$\varphi (F)=A^{F}(\lambda_{PR})/A_{550}^{F}$$

where $A^{F}(\lambda_{PR})$ is the extinction of particles at LPR wavelength $\lambda_{PR}$ after PM treatment with the fluence value F; $A_{550}^{F}$ is the extinction value at the wavelength of the short-wavelength maximum in the region of 500–600 nm (if it exists), or is the extinction value at the wavelength of 550 nm, if there is no maximum in this region. Note that the value of the φ parameter can vary within relatively wide limits, depending on the specific sample and type of particle. To quantify PM, we introduce the PM factor $\eta_{PM}$:

(2) $$\eta_{PM}=\frac{\varphi (0)-\varphi (F)}{\varphi (0)-\varphi (F_{\max})}$$

where $F_{\max}$ is the maximum fluence used in PM experiments. The PM factor has the meaning of the fraction of photomodified nanoparticles. Unlike the φ parameter, the $\eta_{PM}$ factor has a universal value range from 0 to 1 (100%) regardless of the particle shape, size, and structure. The $\eta_{PM}$ factor was calculated and plotted depending on the value of laser fluence for 4 types of gold nanoparticles (Figure 10), as well as for particles coated with a silica shell (Figure 11).

Sometimes, it is not possible to carry out the entire series of PM experiments to ensure complete PM as indicated by the limiting value $\eta_{PM}\to 1$. In this case, an approximate PM factor can be used. At maximal fluence, the PM parameter $\varphi (F_{\max})$, the corresponding proportion of the initial particles in the sample, is close to zero. Then Equation (2) can be simplified to

(3) $$\eta_{PM}^{a}=1-\frac{\varphi (F)}{\varphi (0)}$$

This equation can be used even if the maximal fluence under specific experiments did not produce a complete PM of particles. In Figure 10 and Figure 11, we compare the calculations with Equation (2) (black lines) and Equation (3) (red lines).

As can be seen from Figure 10 (black lines), the following regularity applies to all particles. At a minimal fluence, the $\eta_{PM}$ factor increases slowly. Then, there is a rather sharp, rapid growth, after which the value of the $\eta_{PM}$ factor reaches saturation. For a specific nanoparticle type, the boundary values of these transitions are different, but the general trend remains unchanged. Gold nanorods are quite sensitive to PM (Figure 10a, black line), and a sharp increase in $\eta_{PM}$ starts if the fluence reaches 13 mJ/cm^2^ and ends at 130 mJ/cm^2^. Further growth becomes slower, and the function reaches a plateau. Complete PM of nanoparticles occurs at a fluence of 2080 mJ/cm^2^ when the PM factor $\eta_{PM}$ reaches its maximum value. For AuNSTs (Figure 10b, black line), a noticeable PM begins with a threshold RE value of 130 mJ/cm^2^, and a sharp increase in the $\eta_{PM}$ factor is observed up to a maximum RE value of 520 mJ/cm^2^. For AuNAs and fluence values from 13 to 65 mJ/cm^2^, a gradual increase in the $\eta_{PM}$ factor is observed (Figure 10c, black line). With a further increase in RE from 65 to 260 mJ/cm^2^, this increase becomes sharper and leads to the maximum $\eta_{PM}$ value at RE equal to 520 mJ/cm^2^. AuNSHs begin to be modified at fluence values equal to 65 mJ/cm^2^, while the $\eta_{PM}$ factor shows a sharp increase (Figure 10d, black line). After reaching a value of 260 mJ/cm^2^, the rapid growth slows down and the $\eta_{PM}$ value is close to 1 when fluence reaches 520 mJ/cm^2^.

Coating with a silica shell increases the resistance of gold nanorods to laser radiation and increases their PM thresholds (Figure 11, black curve) compared to the original nanorods without a shell (Figure 10a). For a minimal silica shell thickness (24 nm, (Figure 11a), a very slow increase in $\eta_{PM}$ is observed at low levels of fluence. A significant increase in the $\eta_{PM}$ parameter is observed when the fluence value exceeds the value of 65 mJ/cm^2^. Rapid growth occurs up to a fluence of 2080 mJ/cm^2^, after which the growth becomes smoother and reaches a maximum at a fluence of 4800 mJ/cm^2^. For two samples with a thicker shell (30 and 57 nm), the $\eta_{PM}$ factor behaves similarly. At low fluences, the particles are stable under laser irradiation, and when the value 40 mJ/cm^2^ is reached, the $\eta_{PM}$ factor begins to rapidly increase. This growth continues until the fluence reaches 2080 mJ/cm^2^, and the PM parameter reaches its maximum value at 4800 and 5300 mJ/cm^2^ for shell thicknesses of 30 and 57 nm, respectively. Table 1 summarizes the fluence values, corresponding to the initial and complete PM of bare Au nanoparticles and nanocomposites AuNR@SiO_2_. It was also found that the order of distribution of nanoparticles according to the threshold for the onset of photomodification is in good agreement with the order of their distribution according to the ratio of surface area to volume, which is quite reasonable.

Thus, based on the dependence of the PM factor on fluence, we arrive at the same conclusions that were already formulated earlier when analyzing the TEM images and extinction spectra. First, the deposition of a complete silica shell of an arbitrary thickness (from 24 to 57 nm) leads to a significant increase in the stability of nanoparticles and an increase in their PM threshold. In particular, when AuNRs are covered with a silica shell, an increase in the threshold for the onset of PM is observed from 13 to 65–130 mJ/cm^2^, i.e., by a factor of 5–10. The threshold for complete PM also increases significantly. The second important conclusion is that PM is almost independent of the thickness of the silica shell. From a practical point of view, this leads to a universal recipe for enhancing the photostability of Au nanoparticles through the covering by a thin silica shell without precisely adjusting its geometry and laser irradiation parameters.

## 4. Conclusions

In this work, we have studied the morphological changes induced by nanosecond single-pulse laser irradiation of gold nanoparticles of various shapes and structures, including those coated with a silica shell. Laser exposure with the particle LPR wavelength of 900 nm was carried out during the movement of nanoparticles in a capillary. The single-shot regime was obtained by adjusting the capillary flow and pulse repetition rate. By combining the TEM examination with extinction spectra measurement under variable laser fluence, we found two threshold fluence values corresponding to the initial and complete photomodification of original particles into polydisperse sphere ensembles. For the quantitative assessment of the PM degree, we introduced a new quantitative parameter called the PM factor, which was calculated in terms of the normalized extinction spectra.

From TEM data and extinction spectra, the initial PM fluence threshold was found to increase in the series of nanoparticles: nanorods—nanoantennas—nanoshells—nanostars. The fluence threshold for the complete transformation of particles into spheres increases in the order: nanoshells—nanostars and nanoantennas—nanorods.

The photostability of AuNRs was strongly increased by coating with a thin 24 nm silica layer. Specifically, the threshold value for the beginning of PM was increased by a factor of five compared to the initial rods and the fluence threshold for the complete transformation of particles was increased by a factor of two. For AuNRs coated with 24, 30, and 57 nm silica shells, the onset of the PM threshold does not depend on the silica shell and equals 65 mJ/cm^2^.

In summary, our results provide important quantitative information for the design of biomedical experiments with plasmonic particles irradiated by nanosecond laser pulses. The proposed spectral assessment of PM can be used for the selection of optimal synthesis protocols and irradiation regimes that ensure the retention of nanoparticle plasmonic properties after single-pulse laser irradiation.

## Figures and Tables

**Table 1:** Fluence threshold values for the beginning and end of photomodification for various types of gold and silica-coated gold nanoparticles, obtained from extinction spectra.

Sample	Fluence at the Initial PM (mJ/cm^2^)	Fluence for Complete PM (mJ/cm^2^)
AuNAs	13	520
AuNRs	13	2080
AuNSH	65	520
AuNST	130	1040
AuNR@SiO_2_-24 nm	65	4800
AuNR@SiO_2_-30 nm	65	4800
AuNR@SiO_2_-57 nm	65	5430

## Data Availability

The data presented in this study are available on request from the corresponding author.
